# Are Child's Class C Patients With Acute Variceal Bleeding Worth Treating?

**DOI:** 10.1155/1991/46354

**Published:** 1991

**Authors:** G. W. Johnston, E. F. A. Spencer, F. J. Mullan

**Affiliations:** Royal Victoria Hospital Grosvenor Road N. Ireland Belfast BT12 6BA UK

## Abstract

In the ten year period January 1980 to December 1989, 102 patients with Child’s Class C liver disease (Pugh's Modification) were admitted with acute variceal bleeding to one surgical unit with a policy of early sclerotherapy. There were 56 males and 46 females; the average age was 55 years (range 28–77). Fifty-three suffered from alcoholic cirrhosis. Four died before definitive treatment could be carried out, three from liver failure and one from uncontrolled bleeding. Of the remaining 98 patients, eight had urgent oesophageal transection with three deaths from hepatorenal failure; 90 had sclerotherapy with 19 hospital deaths, nine from recurrent bleeding, eight from liver failure often coupled with renal failure and two from respiratory complications. Of the 76 who survived to leave hospital, 52 received chronic injection sclerotherapy, 10 had elective oesophageal transection and 14 did not have further elective intervention for various reasons. Surviving patients have been followed up at a special Liver Clinic with minimum follow up of one year. Although no patient has yet survived ten years, the one, five and eight year survivals of 50%, 21% and 13% suggest that salvage of thdse patients is worthwhile.


*HPB Surgery*, 1991, Vol. 4, pp 271–276
Reprints available directly from the publisher
Photocopying permitted by license only

© 1991 Harwood Academic Publishers GmbH
Printed in the United Kingdom

### ARE CHILD'S CLASS C PATIENTS WITH ACUTE VARICEAL BLEEDING WORTH TREATING?

G.W. JOHNSTON, E.F.A. SPENCER and F.J. MULLAN
*Royal Victoria Hospital, Belfast, UK*

*(Received 13 February 1991)*

In the ten year period January 1980 to December 1989, 102 patients with Child's Class C liver disease (Pugh's Modification) were admitted with acute variceal bleeding to one surgical unit with a policy of early sclerotherapy. There were 56 males and 46 females; the average age was 55 years (range 28–77). Fifty-three suffered from alcoholic cirrhosis. Four died before definitive treatment could be carried out, three from liver failure and one from uncontrolled bleeding. Of the remaining 98 patients, eight had urgent oesophageal transection with three deaths from hepatorenal failure; 90 had sclerotherapy with 19 hospital deaths, nine from recurrent bleeding, eight from liver failure often coupled with renal failure and two from respiratory complications. Of the 76 who survived to leave hospital, 52 received chronic injection sclerotherapy, 10 had elective oesophageal transection and 14 did not have further elective intervention for various reasons. Surviving patients have been followed up at a special Liver Clinic with minimum follow up of one year. Although no patient has yet survived ten years, the one, five and eight year survivals of 50%, 21% and 13% suggest that salvage of these patients is worthwhile.

KEY WORDS: Oesophageal varices, Child's Class C patients, sclerotherapy, oesophageal transection

### INTRODUCTION

It is established that in patients with variceal bleeding the most important factor influencing short and long term survival is the degree of liver failure. Pugh's modification of Child's classification, based on clinical and biochemical parameters, gives a good working assessment of liver dysfunction. In Child's Class C patients, the mortality for the index bleed is high and the risk of rebleeding within five days of the admission haemorrhage is 63 per cent[1]. It is therefore essential that active therapy is initiated as soon as possible after the initial bleed. However some take a very pessimistic view and consider that all the therapeutic regimes available "give only short lived benefits and do not affect long term survival"[2]. We therefore looked at a consecutive series of 102 Child's Class C patients with acute variceal bleeding admitted to one unit over a ten year period.

### PATIENTS

Between January 1980 and December 1989, 102 patients with Child's Class C liver disease (Pugh's modification) were admitted with acute variceal bleeding to one

Address correspondence to: G.W. Johnston, Mch FRCS, Wards 15/16, Royal Victoria Hospital, Grosvenor Road, Belfast BT12 6BA N. Ireland, UK

surgical unit with a policy of early sclerotherapy. These 102 patients represent 51 per cent of the total patients admitted to that unit with acute variceal bleeding. All patients have been followed up for a minimum of one year or until death. There were 56 males and 46 females; the average age was 55 (range 28–77). The aetiology of liver disease is given in Table 1; 52 per cent were alcoholic in origin.

**Table 1** Aetiology of liver pathology

| | |
|---|---|
| Alcoholic | 53 |
| Cryptogenic | 23 |
| Chronic Active Hepatitis/Cirrhosis | 10 |
| Primary Biliary Cirrhosis | 7 |
| Secondary Biliary Cirrhosis | 2 |
| Haemochromatosis | 2 |
| Budd Chiari Syndrome | 2 |
| Sarcoidosis | 1 |
| Cystic Fibrosis | 1 |
| Toxoplasmosis | 1 |

Of the 102 patients, four died after admission before definitive treatment could be carried out, three from liver failure and one from uncontrolled bleeding. Of the remaining 98 patients, eight had urgent oesophageal transection with three post-operative deaths due to hepatorenal failure. The remaining 90 patients proceeded to sclerotherapy. In 79, this was done as an urgent procedure within 24 hours of the onset of bleeding. In these patients there were 19 hospital deaths, nine from recurrent bleeding, eight from liver failure, often coupled with renal failure, and two due to respiratory complications. Fifteen of these patients required two injections for control of bleeding during the admission and five of the 19 deaths were in this group. Two patients required three injections and both survived. Eleven patients had non-emergency sclerotherapy without mortality during this first admission; three of these patients did not receive any further sclerotherapy. Of the 76 patients who survived to leave hospital nine further patients had no further interference for various reasons. Ten went on to elective oesophageal transection with two hospital deaths. One of these elective transection patients continued to drink and rebled from varices four years after operation; bleeding was controlled by sclerotherapy.

Fifty seven patients were entered into a chronic injection programme but in fact five patients never received any chronic injections. These five were readmitted with acute bleeding and received a total of fourteen acute injections during twelve admissions. The remaining 52 patients in the chronic injection group received a total of 144 repeat injections; the average number of chronic injections was 2.8 and the range one to eight. During the whole period under review, 18 patients on the chronic injection programme required 31 readmissions with further bleeding before variceal obliteration was obtained or death ensued. Sclerotherapy controlled the bleeding in 27 of the 31 admissions.

Currently only 21 per cent of the series remain alive. The one, five and eight year survivals are 50 per cent, 21 per cent and 13 per cent respectively. No patient has yet survived ten years. The majority of the 55 late deaths in the eleven year follow up period were due to liver failure (Table 2). Upper GI bleeding was the cause of

**Table 2** Causes of late death

| | |
|---|---|
| Liver Failure | 31 |
| Upper GI Bleeding | 10 |
| Post-operative Bleeding | 2 |
| Sepsis | 2 |
| Myocardial Infarction | 2 |
| Mesenteric Infarction | 1 |
| Pulmonary Embolism | 1 |
| Renal Failure | 1 |
| Respiratory Failure | 1 |
| Cancer | 1 |
| Ulcerative Colitis | 1 |
| Unknown | 2 |
| TOTAL | 55 |

late deaths in 10 patients; eight due to variceal bleeding, one from peptic ulceration and one from gastritis. Two other non-variceal bleeding deaths occurred post-operatively, one following dental extraction and the other after transection and splenectomy.

### DISCUSSION

In cirrhotics with Child's Class C liver disease, variceal bleeding is often a terminal event. The problem is not only the bleeding varix, but also the underlying sickness of the patient's hepatocytes. Thus, although some patients succumb from uncontrolled bleeding, a greater number die from liver failure. In Cello and colleagues' randomized trial of 64 Child's Class C patients with variceal bleeding, 50 per cent of those receiving sclerotherapy survived to leave hospital, compared to only 44 per cent of those treated by shunt surgery[3]. Huizinga and colleagues reported a 54 per cent mortality for oesophageal transection in Child's Class C patients compared to 26 per cent mortality for injection sclerotherapy[4]. It has been suggested that if two episodes of sclerotherapy fail to control bleeding, one should proceed to oesophageal transection[5]. However in Grade C patients, operative intervention after failed sclerotherapy, carries a prohibitive mortality. No Child Grade C patient survived oesophageal transection following failed sclerotherapy in the series reported from Liverpool[6]. Willson and colleagues reported four out of 22 Grade C patients who survived transection after failed sclerotherapy[7]. In our series, eight Grade C patients were treated by emergency transection without prior sclerotherapy; there were three hospital deaths. The five surviving patients were all dead in less than two years; all died from liver or renal failure. Only one experienced recurrent bleeding which was managed by sclerotherapy. Although there were only two deaths in the ten transections done electively, in general we do not favour surgery for these Grade C patients. It should be possible to control acute bleeding with injection sclerotherapy in about 90 per cent of the patients. Where repeated sclerotherapy fails to control the bleeding, it is unlikely that conventional surgery will salvage many of these high risk patients. Where facilities are available, these patients should be considered for urgent liver transplantation[8]. In situations where this is not feasible, persistence with Somatostatin infusions or intermittent tamponade probably gives the best chance of survival.

Our results suggest that it is worthwhile treating these Child's Class C patients. Although there was an initial 25 per cent hospital mortality, 50 per cent were still alive at one year and 21 per cent at five years.

### *References*

1. Burroughs, A.K. (1988) The management of bleeding due to portal hypertension. The management of acute bleeding episodes. *Quarterly Journal of Medicine*, **254**, 447–458
2. Editorial (1981) Why treat cirrhosis? *British Medical Journal*, **283**, 338
3. Cello, J.P., Crass, R. and Trunkey, D.D. (1982) Endoscopic sclerotherapy versus oesophageal transection in Child's Class C patients with variceal haemorrhage. Comparison with results of portacaval shunt. Preliminary report. *Surgery*, **91**, 333–338
4. Huizinga, W.K.J., Angorn, I.B. and Baker, L.W. (1985) Esophageal transection versus injection sclerotherapy in the management of bleeding esophageal varices in patients at high risk. *Surgery, Gynaecology and Obstetrics*, **160**, 539–545
5. Burroughs, A.K., Hamilton, G., Phillips, A., Mezzanotte, G., McIntyre, N. and Hobbs, K.E.F. (1989) A comparison of sclerotherapy with staple transection of the oesophagus for the emergency control of bleeding from oesophageal varices. *New England Journal of Medicine*, **321**, 857–862
6. Jenkins, S.A. and Shields, R. (1989) Variceal haemorrhage after failed injection sclerotherapy: the role of emergency oesophageal transection. *British Journal of Surgery*, **76**, 49–51
7. Willson, P.D., Kunkler, R., Blair, S.D. and Reynolds, K.W. (1989) Is emergency oesophageal transection for uncontrolled bleeding varices worthwhile? *Gut*, **31**, A595
8. McCormick, P.A., Greenslade, L., Cardin, F., Hobbs, K.E.F., McIntyre, N. and Burroughs, A.K. (1990) Oesophageal staple transection (OST) as a salvage procedure following failure of acute injection sclerotherapy. *Gut*, **31**, A1214

*(Accepted by S.Bengmark 15 March 1991).*

### INVITED COMMENTARY

This is a very important paper. It clearly demonstrates that even patients with severe liver disease warrant serious consideration for treatment to prevent their dying as a result of variceal haemorrhage.

The authors report that two of their patients who needed three episodes of injection sclerotherapy survived. They do not, however, tell us for how long. I think it is important to note that this group of patients has an excessively high mortality and both Terblanche's group (Bornman, P.C. *et al.*, [1986] S.Afr.Med.J, 70:34–36) and our group at the Royal Free (Burroughs, A.K. *et al.* [1989] NEJM 321:857–862) have clearly demonstrated this. If we had some way of identifying these patients in advance it could be argued that this is one group, albeit a very small one, in whom any therapy is a waste of time and resources.

It was not clear from the paper why ten of their patients went on to 'elective' oesophageal transection. Many papers have now shown quite clearly that oesophageal transection has its only value in controlling acute haemorrhage. Furthermore we have shown that the mortality of such an approach is no higher than for sclerotherapy of the same group of patients who fail initial conservative treatment and the initial results may be somewhat better. However we are all agreed that alone it does not protect significantly against rebleeding and in many cases only provides a few weeks or months for the patient's general condition to be fully assessed prior to a more definitive form of long-term treatment.

Finally, this paper demonstrates clearly that the long-term life expectancy of these patients with Child's C category of liver disease is limited and related to the severity of their liver disease. Logistically it is impossible to recommend liver transplantation for all patients with liver disease who develop the complication of oesophagogastric variceal haemorrhage but surely this group warrants serious consideration for liver replacement once the initial haemorrhage has been controlled.

K. E. F. Hobbs
Professor of Surgery
Royal Free Hospital
London, UK

### INVITED COMMENTARY

Johnston and co-authors have demonstrated that an aggressive therapeutic regimen consisting of acute and chronic endoscopic variceal sclerosis and/or operative esophageal transection results in salvage of a significant percentage of Child's class C patients with chronic liver disease and variceal hemorrhage. Their approach provided early, one-year, and five-year survival rates of 75%, 50% and 21% respectively. Although the majority of deaths were secondary to hepatic failure, ten of 26 (38%) early mortalities and eight of 55 (15%) late deaths were due to uncontrolled variceal bleeding. Could these results have been improved by widening the spectrum of therapies offered to these high risk patients?

Endoscopic sclerotherapy effectively controls acute variceal hemorrhage in 85 to 90 per cent of patients. Terblanche[1] has shown, however, that when bleeding persists after two treatment sessions, a mortality rate of 90 percent can be expected in Child's class C patients unless operative intervention is undertaken. Child's class C includes a diverse group of patients, some of whom are clearly not salvageable by any means and others with less advanced liver disease who have a reasonable chance of surviving an operation (operative mortality rate of 25% or less). We prefer emergency portal decompression for such patients who have failed sclerotherapy, since there is no convincing evidence that stapled esophageal transection is associated with a lower mortality rate and because a portal systemic shunt provides superior long-term control of variceal bleeding. An emergency shunt should also be performed early in the course of patients with bleeding from gastric varices or portal hypertensive gastropathy. Endoscopic sclerotherapy and stapled esophageal transection are generally ineffective in these groups of patients.

Endoscopic variceal sclerosis is a reasonable, long-term therapeutic option for many Child's class C patients who bleed from esophageal varices. However, in two recent controlled trials of shunt surgery versus sclerotherapy, approximately one-third of patients eventually failed sclerotherapy[2,3]. Greater than one-third of patients in both of these trials were Child's class C. In the Atlanta investigation, most of the patients who failed sclerotherapy were salvaged by surgery[2]. In contrast, the majority of sclerotherapy failures in the Nebraska trial bled to death before they could undergo surgery because they lived in rural areas where urgent surgery for portal hypertension was not available[3]. Based on the results of these trials, we have concluded that elective shunt surgery is preferable to chronic sclerotherapy for noncompliant patients and for individuals living in relatively remote geographic areas.

Another consideration in the definitive treatment of Child's class C variceal bleeders is that Child's class and, therefore, operative risk status can frequently be improved by a prolonged interval of medical management after acute variceal hemorrhage has been controlled by nonoperative therapies. Patients with relatively acute decompensation of functional hepatic reserve secondary to a recent alcohol binge or to transient hypotension are most likely to benefit from this approach. In one recent investigation, fifteen Child's class C patients, in whom acute variceal hemorrhage was controlled by nonoperative means, demonstrated statistically significant improvement in the parameters contributing to Child's classification during a mean medical management interval of 41 days prior to elective surgery[4]. The operative mortality rate of this initially high risk group was only 13 percent.

*References*

1. Terblanche, J. (1984) Sclerotherapy for emergency variceal hemorrhage. *World J. Surg.*, **8**, 653
2. Henderson, J.M., Kutner,M.H., Millikan, W.J., Jr., Galambos, J.T., Riepe, S.P., Brooks, W.S., Bryan, F.C. and Warren, W.D. (1990) Endoscopic variceal sclerosis compared with distal splenorenal shunt to prevent recurrent variceal bleeding in cirrhosis.*Ann.Intern.Med.*, **112**, 262
3. Rikkers, L.F., Burnett, D.A., Volentine, G.D. *et al.* (1987) Shunt surgery versus endoscopic sclerotherapy for long-term treatment of variceal bleeding. Early results of a randomized trial. *Ann.Surg.*, **206**, 261
4. Holman, J.M., Jr. and Rikkers, L.F. (1980) Success of medical and surgical management of acute variceal hemorrhage. *Am.J.Surg.*, **140**, 816

Layton F. Rikkers,
Professor and Chairman,
Department of Surgery
University of Nebraska Medical Center
Omaha, Nebraska, USA

